# Integrated metabolomics and the microbiome reveal the compatibility mechanism of the Suxiao Jiuxin pill in the treatment of stable coronary artery disease

**DOI:** 10.1186/s13020-025-01198-8

**Published:** 2025-09-01

**Authors:** Wanqi Le, Jingyu Liao, Yuhao Zhang, Jingjing Xu, Yuanyuan Zeng, Houkai Li, Xiaoxu Shen, Gaosong Wu, Weidong Zhang

**Affiliations:** 1 Institute of Interdisciplinary Integrative Medicine Research, Shanghai University of Traditional Chinese Medicine, No. 1200 Cai Lun Road, Pudong New District, Shanghai, 201203 China; 2 Dongzhimen Hospital Affiliated to Beijing University of Chinese Medicine, No. 5 Haiyuncang, Dongcheng District, Beijing, 100700 China; 3 School of Pharmacy, Shanghai University of Traditional Chinese Medicine, No. 1200 Cai Lun Road, Pudong New District, Shanghai, 201203 China; 4 School of Pharmacy, Naval Medical University, Shanghai, 200433 China; 5 Institute of Medicinal Plant Development, Chinese Academy of Medical Sciences & Peking Union Medical College, Beijing, 100193 China

**Keywords:** Suxiao Jiuxin pill, Stable coronary artery disease, *Chuanxiong Rhizoma*, Borneol, Compatibility mechanism

## Abstract

**Background:**

The Suxiao Jiuxin pill (SJP) is a Chinese patent medicine that is used for the treatment of stable coronary artery disease (SCAD). However, the compatibility mechanism of SJP in treating of SCAD is still unclear. This study aimed to elucidate the serum metabolic profiles of patients with SCAD treated with SJP and to decipher the compatibility mechanism of its effective components, *Chuanxiong Rhizoma* and borneol.

**Methods:**

We employed metabolomics to assess the serum metabolic profiles of SCAD patients before and after treatment with SJP through metabolomics. Additionally, the compatibility mechanism of the multicomponent pairing of *Chuanxiong Rhizoma* and borneol was explored using metabolomics and 16S rRNA sequencing.

**Results:**

Our findings indicate that SJP significantly modulates lipid metabolism in SCAD patients, with particular impacts on glycerophospholipids and fatty acyls. Coadministration of *Chuanxiong Rhizoma* and borneol in mice demonstrated that borneol increases the absorption of the active components of *Chuanxiong Rhizoma* into the blood in a dose-dependent manner. This effect correlated with the dose-dependent enrichment of *A. muciniphila* and its role in modulating host lipid metabolism (glycerophospholipids and fatty acyls). Moreover, the combination of *A. muciniphila* and *Chuanxiong Rhizoma* also significantly promoted the absorption of the active components of *Chuanxiong Rhizoma* into the blood and affected host lipid metabolism (glycerophospholipids and fatty acyls).

**Conclusion:**

This study is the first to demonstrate a link between SJP treatment in SCAD patients and improved lipid metabolism. Borneol enriches *A. muciniphila* in a dose-dependent manner, thereby regulating host lipid metabolism and facilitating the absorption of the active components of Chuanxiong Rhizoma into the blood.

**Supplementary Information:**

The online version contains supplementary material available at 10.1186/s13020-025-01198-8.

## Introduction

With the accelerated ageing of the global population, cardiovascular disease (CVD) has become a major factor in human health, and the prevalence and mortality of the population are constantly increasing [1, 2]. CVD is the result of a variety of pathological factors that involve a variety of processes, such as cell inflammation, apoptosis, oxidative stress and thrombosis [3–5]. Currently, nitrates, fibrates and statins dominate the landscape of cardiovascular pharmacotherapy. However, owing to the single chemical composition of each of these drug classes, they can act on only a few targets, with a high risk of chemical residues, and long-term use is likely to cause adverse reactions [6–8]. Since the control of CVD by Western medicine has not been satisfactory, clinicians are increasingly using traditional Chinese medicine (TCM) as a supplement and alternative to the primary and secondary prevention of CVD and have achieved good results [9–11].

CVD is predominantly characterized by thrombotic events. Consequently, Chinese patent medicines for promoting blood circulation and removing blood stasis are frequently employed in the clinical management of CAD [12]. The core herbal components, such as *Salvia miltiorrhiza Bunge*, *Chuanxiong Rhizoma*, *Carthamus tinctorius* L. and *Radix Paeoniae Rubra*, are typically integrated with tonic and resuscitation drugs. Among these, borneol features prominently in the formulation of resuscitation drugs [13]. Borneol is known to increase the concentrations of drugs that target CVD, increase the permeability of the blood‒brain barrier, and improve drug targeting and enrichment at pathological sites [14]. As a representative Chinese patent medicine for promoting blood circulation and removing blood stasis, the Suxiao Jiuxin pill (SJP) is composed of borneol and *Chuanxiong Rhizoma*, which has been proven to be effective in treating patients with stable coronary artery disease (SCAD) through clinical trials [15, 16] and can play a cardioprotective role through multiple components and multiple targets [17–19]. Our recent research has underscored the pivotal role of gut microbiota‒host metabolism interactions in the pathogenesis of SCAD [20]. Additionally, SJP significantly ameliorates the gut microbiota in acute myocardial infarction (AMI) model animals and corrects lipid metabolism disorders [21]. Despite these findings, the relationship between the compatibility mechanism of SJP in treating SCAD patients and gut microbiota-host metabolic interactions remains to be elucidated.

**Fig. 1:**
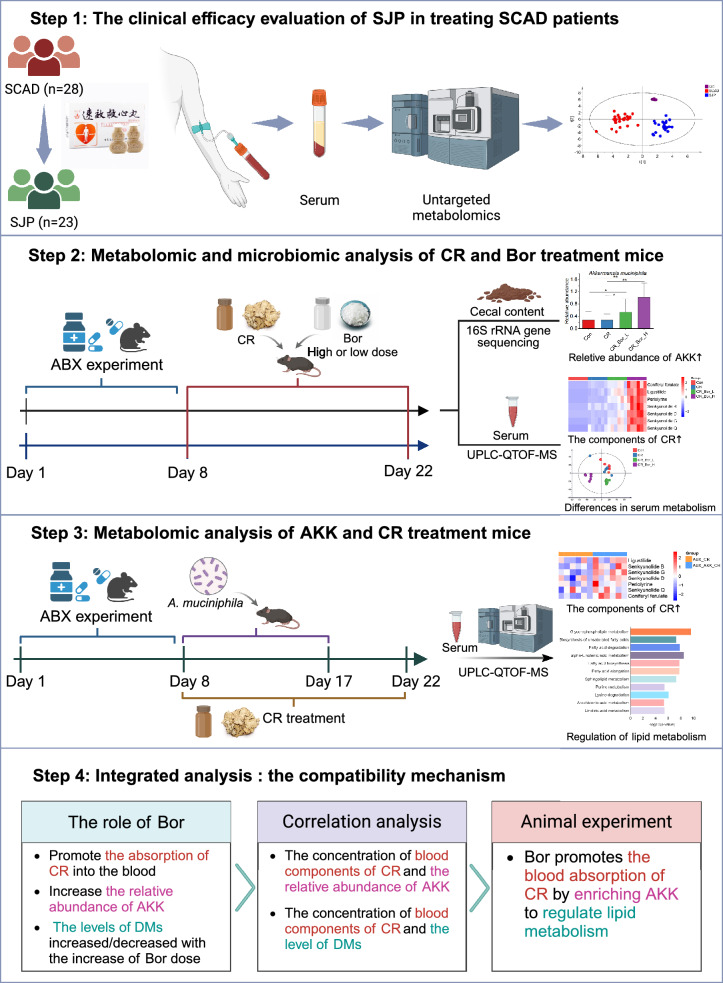
Schematic diagram of the experimental design

In this study, we aimed to reveal the compatibility mechanism of *Chuanxiong Rhizoma*-borneol in the SJP on the basis of the gut microbiome and metabolomics. First, we performed metabolomics analysis on serum samples of patients with SCAD treated with the SJP to determine whether their serum metabolic profiles were significantly affected by the SJP. An antibiotic treatment model was subsequently established to clarify the relationship between the absorption of the active components of Chuanxiong Rhizoma into the blood and the regulation of the gut microbiota by borneol. 16S rRNA sequencing and metabolomics were used to determine the gut microbiota and serum metabolic profiles that were significantly affected by different doses of borneol combined with *Chuanxiong Rhizoma*. Finally, specific gut bacteria that were significantly affected by borneol and *Chuanxiong Rhizoma* were administered to mice, and differences in the blood component concentrations of *Chuanxiong Rhizoma* and the serum metabolic profiles were detected when specific gut bacteria were treated or not (Fig. 1).

## Materials and methods

### Clinical trial design and efficacy evaluation

The clinical trial was conducted in accordance with the Declaration of Helsinki and received ethical approval from the Human Ethics Committees of Dongzhimen Hospital, which is affiliated with Beijing University of Chinese Medicine. The participants provided written informed consent prior to their inclusion in the study. We enrolled a total of 28 patients with SCAD from August 2018 to December 2019, with detailed inclusion and exclusion criteria described in our previous publications [22].

All participants received standard care as recommended by the European Society of Cardiology and the World Heart Federation. SCAD patients were administered SJP (batch number: 618356) orally at a dosage of 480 mg/day for a continuous period of 2 weeks, and the method for evaluating its efficacy was based on our previous publications [22], detailed efficacy evaluation methods can be found in the supplementary materials. The components of SJP were analysed using liquid chromatography‒mass spectrometry (LC‒MS) and gas chromatography‒mass spectrometry (GC‒MS), with the methodology and identified components detailed in our previous publications [21]. Serum samples were collected from participants at baseline and 2 weeks post-SJP treatment, and the samples were collected by medical staff in a clinical setting within the hospital. The samples were aliquoted and stored at − 80 °C for subsequent analysis.

### Materials and chemicals

LC‒MS-grade acetonitrile, methanol, and formic acid were procured from Fisher Scientific (Fair Lawn, NJ, USA). Ultrapure water, exhibiting a resistivity of 18.2 MΩ, was produced using a Milli-Q water purification system from Millipore (MA, USA). Sodium carboxymethyl cellulose (CMC-Na) and sodium pentobarbital were sourced from the Sinopharm Chemical Reagent Co., Ltd. (Shanghai, China). Chopped meat carbohydrate broth was supplied by Shandong Tuopu Biol-engineering Co., Ltd. (Shandong, China). *Chuanxiong Rhizoma* (batch number: B26040) and borneol (batch number: S47379) were acquired from Shanghai Yuanye Bio-Technology Co., Ltd. (Shanghai, China). Antibiotics, including neomycin sulfate, streptomycin sulfate, penicillin G, and metronidazole, were obtained from Macklin (Shanghai, China). Vancomycin was imported from Adamas (Switzerland).

### Animal experimental design

All the mice utilized in this study were 5-week-old male C57BL/6J mice procured from Shanghai Jihui Laboratory Animal Care Co., Ltd. (Shanghai, China). They were housed and bred in a specific pathogen-free (SPF) environment at the Laboratory Animal Centre of Shanghai University of Traditional Chinese Medicine (Shanghai, China). All the animal experiments adhered to protocols approved by the Committee on the Ethics of Animal Experiments at Shanghai University of Traditional Chinese Medicine (approval number: PZSHUTCM2306250005, dated June 25, 2023).

For the *Chuanxiong Rhizoma* and borneol treatment experiment, the mice were randomly assigned to four groups (*n* = 6): the control (Con) group, the *Chuanxiong Rhizoma* treatment (CR) group, the CR and low-dose borneol treatment (CR_Bor_L) group, and the CR and high-dose Bor treatment (CR_Bor_H) group. Referring to the dosage used in previous publications [23], the dose for CR is 200 mg/kg/d, the dose for Bor_L is 160 mg/kg/d, and the dose for Bor_H is 320 mg/kg/d. CR and Bor were administered by oral gavage once daily for 2 weeks. For the antibiotic (ABX) treatment experiment, a cocktail consisting of streptomycin sulfate (80 mg/kg/d), penicillin G (80 mg/kg/d), neomycin sulfate (80 mg/kg/d), metronidazole (80 mg/kg/d), and vancomycin (40 mg/kg/d) was administered via gavage daily for 7 days. CR (200 mg/kg/d) and Bor_L (160 mg/kg/d) were administered via gavage once daily for 2 weeks after ABX treatment.

For the *Akkermansia muciniphila* (*A. muciniphila*) treatment experiment. First, *A. muciniphila* (ATCC BAA-835) was cultured anaerobically (90% N_2_, 5% CO_2_, and 5% H_2_) at 37 °C in chopped meat carbohydrate (CMC) broth. The culture mixture was centrifuged (4000 rpm, 15 min) and washed twice with sterile PBS, and *A. muciniphila* was resuspended at a final concentration of 1.5 × 10^10^ CFU/mL and administered to the mice by gavage [24]. After 7 days of ABX treatment, the 12 mice were randomly divided into 2 groups (*n* = 6): the ABX combined with CR treatment group (ABX_CR) and the ABX combined with *A. muciniphila* and CR treatment group (ABX_AKK_CR). CR (200 mg/kg/d) in 0.5% CMC-Na aqueous solution was administered via gavage in both groups, and ABX_AKK_CR group mice were administered *A. muciniphila* suspended in 0.2 mL sterile anaerobic PBS daily for 2 weeks, whereas mice in the ABX_CR group were given an equivalent volume of sterile anaerobic PBS instead.

### LC‒MS analysis

On the basis of our previously established LC‒MS data acquisition model [25], the metabolites and blood components in the clinical and animal serum were collected. Chromatographic separation was conducted using a Waters UPLC system with an ACQUITY UPLC HSS T3 column (2.1 × 100 mm, 1.8 μm) at 40 °C. The mobile phase consisted of 0.1% aqueous solutions of formic acid (A) and acetonitrile (B), with a gradient flow of 0.3 mL/min: 5% B, 0–1 min; 5–100% B, 1–13 min; 100% B, 13–15 min; 100–5% B, 15–15.1 min; and 5% B, 15.1–20 min. The injection volume was 2 μL. Mass spectrometry was conducted using a Waters SYNAPT G2-Si HDMS system. Sample preprocessing, instrument parameter settings, data acquisition, and further data analysis are presented in the Supplementary Materials.

### 16S rRNA sequencing of caecal content microbiota

Microbial DNA extraction from the caecal contents was conducted using an EZNA® soil DNA kit (Omega Biotek, Norcross, GA) according to the manufacturer's protocol. The V3–V4 hypervariable regions of the bacterial 16S rRNA gene were amplified with the primers, 338F and 806R; more detailed information can be found in our previous publication [21].

### Statistical analysis

The data are presented as the means ± standard deviations (SDs). Statistical comparisons between two groups were performed using either Student’s t test for normally distributed data or the Wilcoxon rank-sum test for nonparametric data. For evaluating multiple group comparisons, one-way analysis of variance (ANOVA) was employed for normally distributed data, whereas the Kruskal‒Wallis H test was used for nonnormally distributed data. A p value of less than 0.05 was considered to indicate statistical significance.

## Results

### SJP treatment significantly improved lipid metabolism in patients with SCAD

**Fig. 2:**
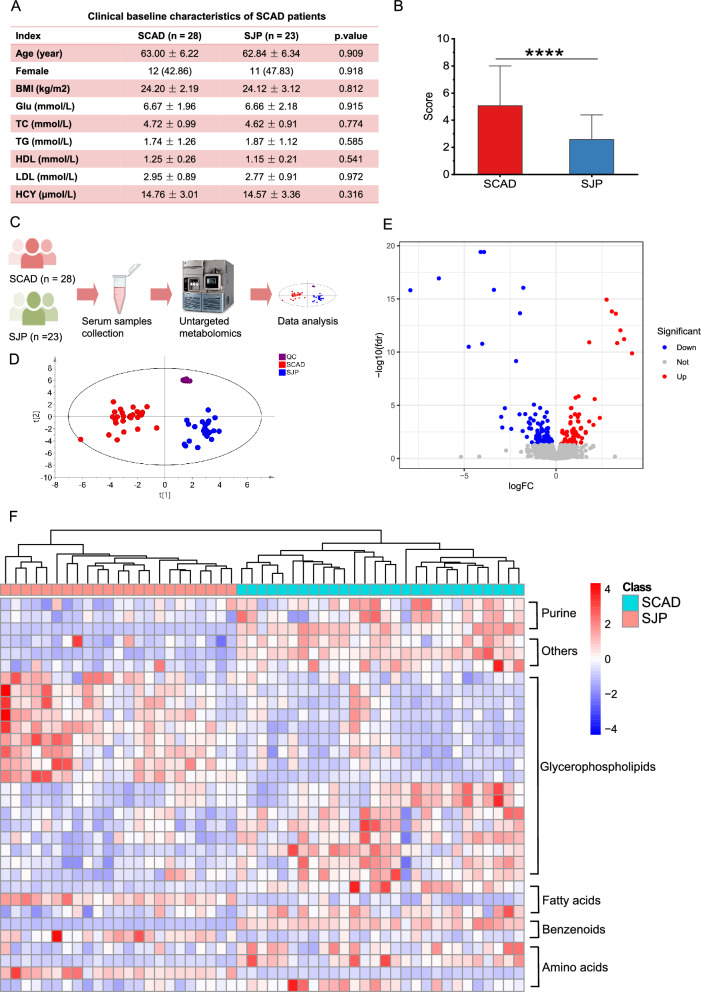
Clinical efficacy evaluation and serum metabolomics analysis of SJP in the treatment of SCAD patients. **A** Clinical baseline characteristics of SCAD patients. **B** The efficacy evaluation index score of SJP treatment for SCAD patients. **C** Flowchart of the clinical serum sample analysis. **D** PCA score plot of the QC, SCAD, and SJP groups. **E** Volcano plot of DMs between the SJP and SCAD groups. **F** Heatmap of DMs with significant changes in the serum. Statistical significance was tested by the two-sided Wilcoxon match-pairs signed-rank test; *, *p* < 0.05; **, *p* < 0.01; ***, *p* < 0.001; ****, *p* < 0.0001

The efficacy of SJP in the treatment of SCAD patients was assessed. We enrolled 28 SCAD patients and administered SJP for 2 weeks. Among these patients, 23 SCAD patients completed the efficacy evaluation posttreatment. The basic clinical characteristics of the participants are depicted in Fig. 2A, with no significant differences observed in any index before or after the administration of SJP. Furthermore, we evaluated the efficacy of SJP in patients with SCAD according to the previous efficacy evaluation method [22]. As shown in Fig. 2B, SJP significantly reduced the symptom scores of SCAD patients, indicating that SJP treatment can markedly alleviate the clinical symptoms associated with SCAD.

We subsequently conducted a metabolomics analysis on the serum samples of SCAD patients treated with SJP/untreated (Fig. 2C). The principal component analysis (PCA) model revealed significant differences in the serum metabolic profiles between SCAD patients treated with SJP and untreated patients (Fig. 2D). After threshold treatment for FDR < 0.05, a total of 184 differential ions (including 78 upregulated and 106 downregulated ions) were obtained (Fig. 2E). Finally, 32 differentially abundant metabolites (DMs) were identified. The relative abundances of these metabolites in the different groups were visualized via a heatmap, which included 17 glycerophospholipids, 4 amino acids, 3 fatty acids, 3 purines, 2 benzenoids, and 3 others (Fig. 2F). Our previous studies also revealed that glycerophospholipids and fatty acids were the most significant types of metabolites resulting from combined treatment with SJP and antibiotics and that the gut microbiota could affect the absorption of the active components of SJP into the blood [21]. The above results indicate that the efficacy of SJP is closely associated with improvements in host metabolism, especially glycerophospholipid and fatty acid metabolism. Moreover, the gut microbiota plays an important role in the efficacy of SJP.

### The gut microbiota is associated with the effect of borneol promoting the absorption of Chuanxiong Rhizoma components into the blood

**Fig. 3:**
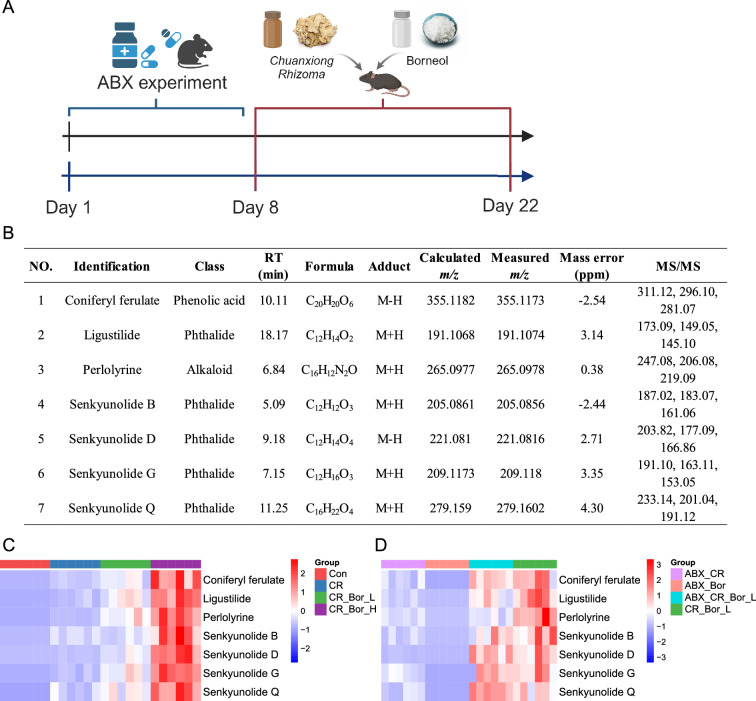
LC‒MS analysis of blood components in *Chuanxiong Rhizoma.* **A** Flowchart of the animal experiments involving *Chuanxiong Rhizoma* and borneol treatment. **B** The effective components of *Chuanxiong Rhizoma* were detected in mouse serum. Heatmap of the absorption of the effective components of *Chuanxiong Rhizoma* into the blood without (**C**) and with (**D**) ABX treatment. Statistical significance was tested by Student’s t test; *, p < 0.05; **, p < 0.01; ***, p < 0.001

Recent studies have shown that borneol can promote the entry of drugs into target organs or tissues through various physiological barriers [14]. To determine whether borneol affects the absorption of *Chuanxiong Rhizoma* components into the blood via the gut microbiota, we conducted an ABX treatment experiment (Fig. 3A). The mice were treated with antibiotics for 1 week to remove the gut microbiota and then treated with *Chuanxiong Rhizoma* and borneol for 2 weeks. Two hours after the last intragastric administration of *Chuanxiong Rhizoma*, serum samples were collected from mice, and the blood components were identified by ultrahigh-performance liquid chromatography with quadrupole time‒flight mass spectrometry (UPLC‒QTOF‒MS). As shown in Fig. 3B, C, by comparison with the self-built database and standards, a total of 7 chemical compounds were identified, including coniferyl ferulate, ligustilide, perlolyrine, senkyunolide B, senkyunolide D, senkyunolide G, and senkyunolide Q. Then, the relative abundances of the above 7 components in each group were visualized (Figs. S1–S8). As shown in Fig. 3D, with increasing borneol dose, the concentrations of the above components gradually increased, suggesting that borneol can promote the absorption of the active components of *Chuanxiong Rhizoma* into the blood in a dose-dependent manner. The clearance of the gut microbiota inhibited the blood absorption of the active components of *Chuanxiong Rhizoma* and inhibited the ability of borneol to promote the blood absorption of the active components of *Chuanxiong Rhizoma*. These results indicate that the ability of borneol to promote the blood absorption of the active components of *Chuanxiong Rhizoma* depends on the gut microbiota.

**Fig. 4:**
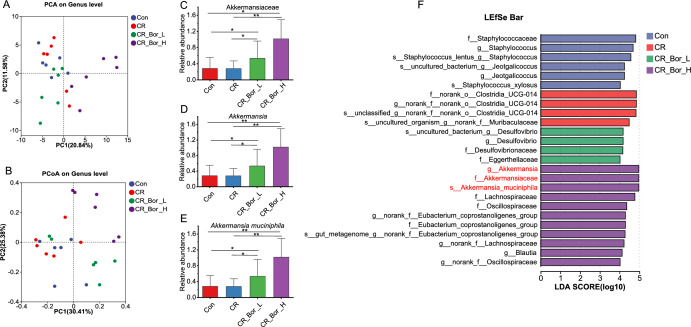
Gut microbiome diversity and structural analysis. **A** PCoA shows the discrimination between the sample groups. **B** PCA shows the discrimination between the sample groups. **C**–**E** Changes in the relative abundances of *Akkermansiaceae*, *Akkermansia*, and *Akkermansia muciniphila* in the Con, CR, CR_Bor_L, and CR_Bor_H groups. **F** Differences in abundance between the Con, CR, CR_Bor_L, and CR_Bor_H groups. Statistical significance was tested by the Wilcoxon rank-sum test; *, p < 0.05; **, p < 0.01;* n* = 6 per group

After confirming that the effect of borneol is associated with the gut microbiota, we examined the bacterial community diversity of the faecal samples via 16S rRNA gene amplicon sequencing. The alpha diversity reflects the richness and diversity of the microbiota. Except for the CR_Bor_H vs. Con group, the changes in the ACE, Chao, Shannon and Simpson indices in the other two groups were not statistically significant compared with those in the Con group (p > 0.05) (Fig. S9A). To measure the degree of similarity between microbial communities, the *β* diversity was further evaluated using the Bray‒Curtis PCA and PCoA models. As shown in Fig. 4A, B, the CR_Bor_H group was significantly separate from the Con group, and there was also a certain trend showing separation between the CR_Bor_L, CR and Con groups. At the phylum level, Firmicutes, Bacteroidetes and Verrucomicrobiota were predominant (Fig. S9B). Interestingly, we found that *A. muciniphila* was considerably enriched at the family–genus–species level and that *A. muciniphila* was enriched in a dose dependent maner by borneol (Fig. 4C–E). LDA and LEfSe were performed to identify the core taxa that best explained the differences between groups. As shown in Fig. 4F, *A.* *muciniphila* was significantly enriched in the CR_Bor_ H group at the family‒genus‒species level. The above results suggest that *A. muciniphila* may play an important role in the process by which borneol promotes the absorption of the active components of *Chuanxiong Rhizoma* into the blood.

### Chuanxiong Rhizoma-borneol treatment significantly affects the serum lipid metabolism in mice

**Fig. 5:**
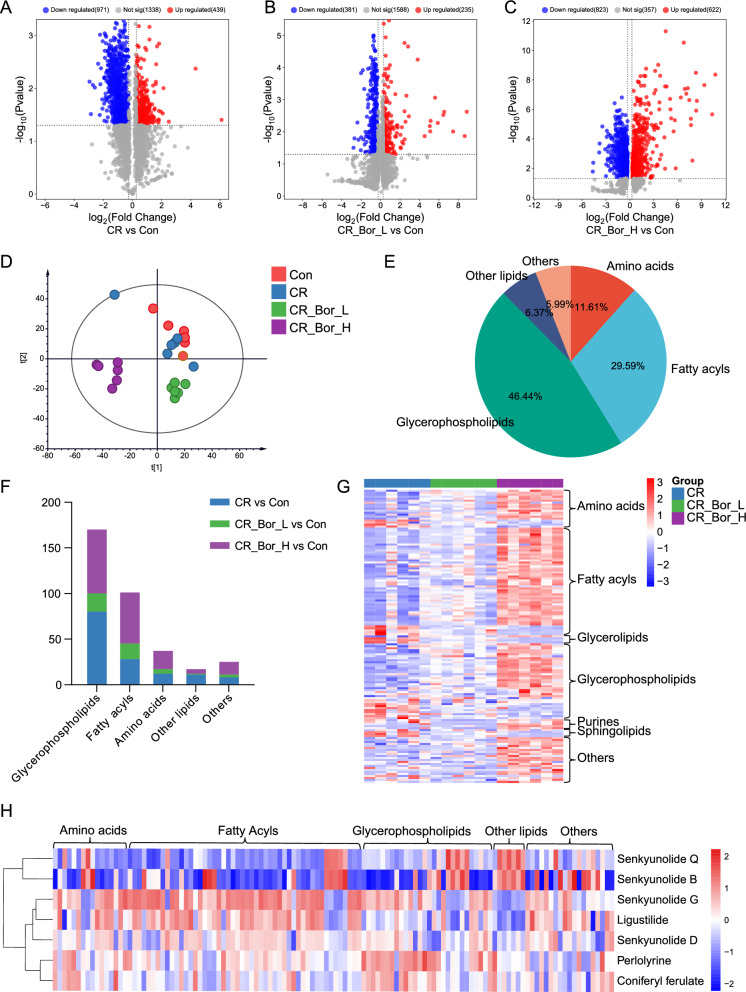
Volcano plots of DMs between the CR and Con groups (**A**), the CR_Bor_L and Con groups (**B**) and the CR_Bor_H and Con groups (**C**). **D** PLS-DA score plots of the Con, CR, CR_Bor_L, and CR_Bor_H groups. **E** Categories and percentages of all DMs. **F** The categories and number of DMs were obtained from the CR, CR_Bor_L, and CR_Bor_H groups. **G** Heatmap of Borneol dose-dependently related DMs in the CR, CR_Bor_L, and CR_Bor_H groups. **H** Correlation analysis of the serum effective constituents of *Chuanxiong Rhizoma* and borneol dose-dependently related to DMs

The gut microbiota can influence host health through the metabolism. Therefore, we further performed metabolomics analysis on the serum samples of the mice in each group. First, the PCA score plot based on the obtained serum metabolomics data revealed that the QC samples were clustered together, suggesting that the instrument had good stability and that the obtained data were accurate and reliable (Fig. S10). On the basis of the screening criteria (p < 0.05 and FC > 1.2), we identified 1410 differentially abundant metabolites (DMs) in the CR group, of which 439 DMs were upregulated and 971 DMs were downregulated compared with those in the Con group (Fig. 5A). Similarly, there were 616 DMs in the CR_Bor_L group, of which 235 DMs were upregulated and 381 DMs were downregulated compared with those in the Con group (Fig. 5B). Compared with those in the Con group, in the CR_Bor_H group, 1445 DMs, including 622 DMs, were upregulated, and 823 DMs were downregulated (Fig. 5C). Using partial least squares discriminant analysis (PLS-DA), we observed distinct clustering of metabolites among the Con, CR, CR_Bor_L, and CR_Bor_H groups; in particular, the separation trend of CR_Bor_H from the other groups was the most obvious (Fig. 5D). A comparison of the MS/MS spectra of the DMs revealed that 234 DMs were ultimately identified; these DMs were mainly glycerophospholipids, fatty acyls and amino acids, accounting for 46.44%, 29.59% and 11.61% of the total, respectively (Fig. 5E). Interestingly, the most significant metabolites in the CR and CR_Bor_H groups were glycerophospholipids and fatty acyls (Fig. 5F).

The absorption of the active components of *Chuanxiong Rhizoma* into the blood is dose dependent, and we speculate that this effect is related to the impact of borneol on the lipid metabolism of the host. We analysed the DMs in a dose-dependent manner and reported that the levels of 121 DMs increased with increasing borneol dose and that the levels of 26 DMs decreased with increasing borneol dose. A heatmap based on their relative contents is shown in Fig. 5G, from which it can be seen that the metabolites with a dose dependency on borneol are still mainly fatty acyls and glycerophospholipids. We further analysed the 147 DMs mentioned above for correlations with the blood components of *Chuanxiong Rhizoma* and found that 131 DMs were significantly correlated with the blood components of *Chuanxiong Rhizoma*. The levels of 7 blood components, namely, perlolyrine, senkyunolide G, senkyunolide D and E-ligustilide, were positively correlated with the levels of DMs (Fig. 5H). These results suggest that borneol promotes the absorption of the active components of *Chuanxiong Rhizoma* into the blood and is closely related to lipid metabolism.

### A. muciniphila promotes the absorption of the active components of Chuanxiong Rhizoma into the blood by affecting host lipid metabolism

**Fig. 6:**
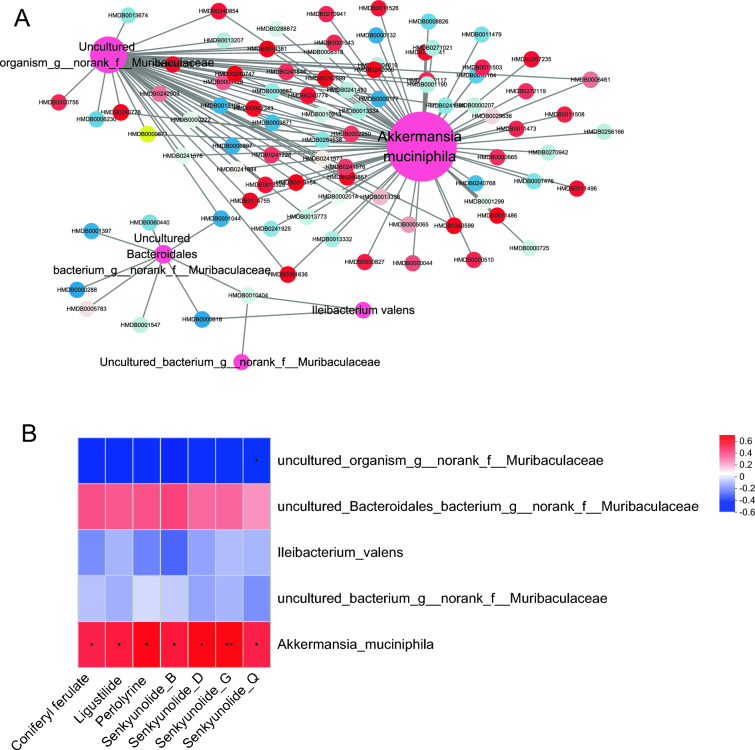
Correlation analysis between DMs in a dose-dependent manner and borneol and the gut microbiota (**A**) and *Chuanxiong Rhizoma* blood components (**B**)

Our study revealed that borneol could enrich *A. muciniphila* in a dose-dependent manner. To clarify the mechanism by which *A. muciniphila* promotes the absorption of the active components of *Chuanxiong Rhizoma* into the blood, the correlations between the top 5 differential bacteria and DMs and the blood components of *Chuanxiong Rhizoma* were analysed. As shown in Fig. 6A, a total of 73 DMs were significantly correlated with *A. muciniphila*, 51 DMs were significantly correlated with *uncultured_organism_g_norank_f_Muribaculaceae*, A total of 8 DMs were significantly correlated with *uncultured_Bacteroidales_bacterium_g_norank_f_Muribaculaceae*, 2 DMs were significantly correlated with *Ileibacterium valens*, and 1 DM was significantly correlated with *uncultured_bacterium_g_norank_f_Muribaculaceae*. Among them, the DMs that were significantly related to *A. muciniphila* were glycerophospholipids and fatty acids. In addition, we analysed the correlations between the 7 blood components of *Chuanxiong Rhizoma* and bacteria and found that all the components presented different degrees of correlation with *A. muciniphila* (Fig. 6B). The above results suggest that *A. muciniphila* may promote the absorption of *Chuanxiong Rhizoma* components into the blood by regulating host lipid metabolism.

**Fig. 7:**
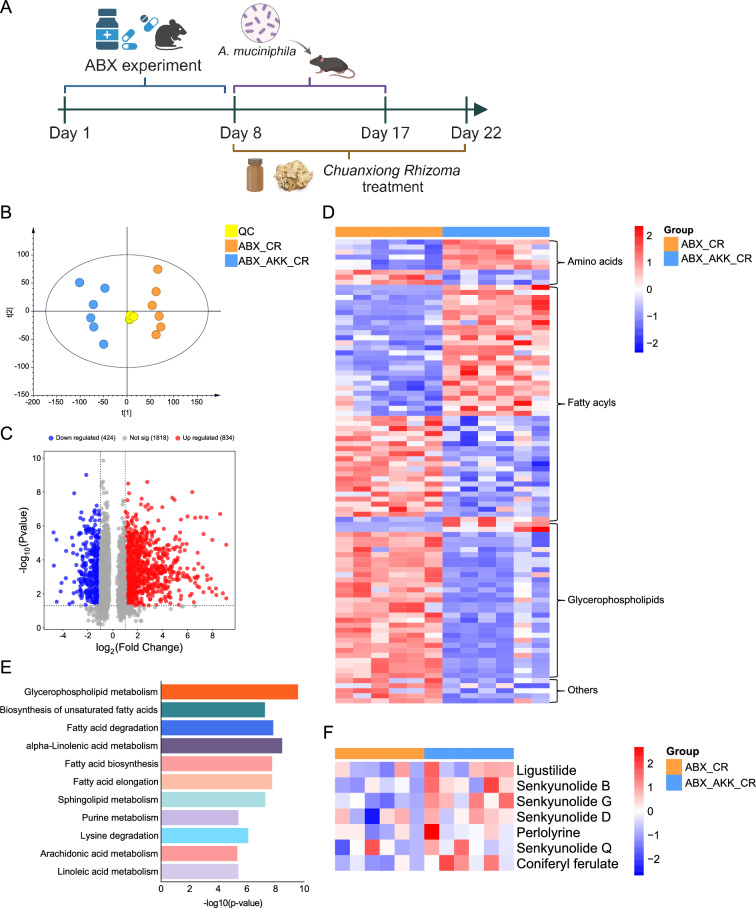
Analysis of metabolites and blood components after combined administration of *A. muciniphila* bacteria and *Chuanxiong Rhizoma*. **A** Flowchart of the animal experiments. **B** PCA score plot of the QC, ABX_CR, and ABX_AKK_CR groups. **C** Volcano plot of DMs between the ABX_AKK_CR and ABX_CR groups. **D** Heatmap of DMs in the ABX_CR and ABX_AKK_CR groups. **E** Changes in metabolic pathways induced by AKK (ABX_AKK_CR vs. ABX_CR) in the serum. **F** Heatmap of the absorption of the effective components of *Chuanxiong Rhizoma* into the blood without and with *A. muciniphila* treatment

To further clarify whether *A. muciniphila* can promote the absorption of the active components of *Chuanxiong Rhizoma* into the blood, we conducted a combined intervention experiment involving *A. muciniphila* and *Chuanxiong Rhizoma*. First, we orally administered ABX to the mice for 7 consecutive days to deplete the gut microbiota, followed by continuous gavage of *A. muciniphila* and *Chuanxiong Rhizoma* for 2 weeks (Fig. 7A). PCA revealed that the serum metabolic profiles of the ABX_CR group and ABX_AKK_CR group were significantly different, suggesting that there were significant differences in the serum metabolomes of these mice (Fig. 7B). The volcano map further revealed that there were 1258 differential ions in the two groups, including 834 upregulated and 424 downregulated ions (Fig. 7C). Based on the HDMB, LIPIDMAPS, and KEGG online database, a total of 92 DMs were identified, which were mainly fatty acyl and glycerophospholipids (Fig. 7D). Further pathway analysis revealed that lipid metabolism pathways, such as glycerophospholipid metabolism, biosynthesis of unsaturated fatty acids, and fatty acid degradation, were significantly enriched (Fig. 7E). We also used LC‒MS to analyse the concentrations of the blood components of *Chuanxiong Rhizoma* in the serum. The combined treatment of *A. muciniphila* and *Chuanxiong Rhizoma* significantly increased the blood absorption concentrations of the active components of *Chuanxiong Rhizoma* (Fig. 7F). The above results indicate that *A. muciniphila* can promote the absorption of the active components of *Chuanxiong Rhizoma* into the blood and that this effect is closely associated with the regulation of host lipid metabolism.

## Discussion

With the acceleration of the ageing processes of the global population, the incidence of cardiovascular disease, which has become the main cause of human death, is increasing [1, 26]. In China, SJP, a commonly used Chinese patent medicine for cardiovascular disease, has been shown to significantly improve the outcomes of cardiovascular disease. For example, SJP can effectively alleviate the symptoms of patients with SCAD. Moreover, as an adjuvant drug, combining it with conventional treatments can improve the efficacy and quality of life of patients with SCAD, and there are no obvious adverse reactions [15]. However, the compatibility mechanism of the effective components of SJP is not clear.

First, we administered SJP to patients with SCAD for 2 weeks and reported that SJP significantly improved various clinical symptoms in patients with SCAD. A further serum metabolomic analysis revealed that SJP could significantly affect the lipid metabolism profiles of SCAD patients, especially the glycerophospholipid and fatty acyl profiles. Lipids, especially glycerophospholipids and fatty acyls, are key contributors to atherosclerosis and are potential pathological factors in cardiovascular diseases [27, 28]. In our previous studies, we also reported that SJP could exert cardioprotective effects in rats with myocardial infarction by remodelling the gut microbiota and host fatty acid metabolism [21]. Notably, the gut microbiota can both transform and synthesize lipids and breakdown dietary lipids to generate secondary metabolites with host modulatory properties, which has a potential impact on the health and physiology of the host [29]. The interaction between the gut microbiota and lipid metabolism affects the formation of atherosclerotic plaques and is a key pathological basis for the development of SCAD [30, 31]. Therefore, SJP may exert its cardioprotective effect by restoring lipid metabolism disorders in SCAD patients, and this effect may be mediated by the gut microbiota. However, it is unclear how borneol and *Chuanxiong Rhizoma* in SJP synergistically affect lipid metabolism.

To clarify the mechanism by which SJP regulates lipid metabolism, we designed an ABX treatment experiment and detected the differences in the absorption of the active components of *Chuanxiong Rhizoma* into the blood with or without ABX treatment. A total of 7 components of *Chuanxiong Rhizoma* (including coniferyl ferulate, E-ligustilide, perlolyrine, senkyunolide B, senkyunolide D, senkyunolide G, and senkyunolide Q) were identified in the serum by LC‒MS, and borneol promoted the absorption of these 7 components into the blood in a dose-dependent manner. However, after ABX treatment, this effect of borneol was eliminated, suggesting that borneol promotes the absorption of the active components of *Chuanxiong Rhizoma* into the blood-dependent gut microbiota. By 16S rRNA sequencing of faecal samples, we found that borneol could enrich *A. muciniphila* in a dose-dependent manner. The intestinal barrier is crucial for maintaining intestinal homeostasis and metabolism, and *A. muciniphila* plays an important role in regulating the intestinal barrier as well as other homeostatic and metabolic functions [32, 33]. *A. muciniphila* can affect intestinal permeability through derived extracellular vesicles to protect the intestinal barrier [34, 35] and maintain intestinal homeostasis through secreted bacterial proteins [36]. Studies have shown that borneol can expand the intercellular space and increase intestinal permeability, promote drug absorption and target organ enrichment, and ultimately achieve enhanced efficacy [37]. Therefore, we speculate that borneol affects the intestinal barrier by enriching *A. muciniphila*, thereby promoting the blood absorption of the active components of *Chuanxiong Rhizoma*.

Next, we analysed the effects of borneol on the DMs in a dose-dependent manner. A total of 147 DMs were dose-dependent with respect to borneol, mainly fatty acyl and glycerophospholipids. Interestingly, these DMs were significantly correlated with the active components of *Chuanxiong Rhizoma* and *A. muciniphila*, and we speculate that *A. muciniphila* may promote the absorption of the active components of *Chuanxiong Rhizoma* into the blood by regulating lipid metabolism. To verify our hypothesis, we administered *A. muciniphila* to mice after the gut microbiota was removed by ABX. The serum metabolic profiles of *A. muciniphila* in mice were significantly different from those in mice not administered ABX, and the main DMs were glycerophospholipids and fatty acyls. Moreover, the administration of *A. muciniphila* significantly promoted the absorption of the active components of *Chuanxiong Rhizoma* into the blood. Studies have shown that *A. muciniphila* monocolonization decreases the expression of CD36 in the jejunum, improves glucose metabolism, affects the levels of multiple fatty acids in plasma, and inhibits vascular calcification through derived fatty acids [38, 39]. Moreover, *A. muciniphila* can promote angiogenesis by reducing intestinal permeability and inflammation [40]. Previous studies have confirmed that borneol can dilate blood vessels and inhibit platelet aggregation [14]. We hypothesize that borneol, by enriching *A. muciniphila*, not only facilitates the absorption of the active components of *Chuanxiong Rhizoma* into the blood but also may directly affect platelets through derived lipids, thereby jointly inhibiting platelet activation. However, the specific mechanisms require further investigation.

However, our research also has several limitations. First, the number of clinical SJP samples used for the treatment of SCAD patients was limited, which precluded the identification of potential lipid biomarkers for efficacy evaluation. Additionally, we did not determine the specific lipid biomarkers that were affected by borneol-enriched *A. muciniphila* by targeted lipidomics. Nevertheless, our previous publications demonstrated that this method can reliably and accurately identify metabolic biomarkers, more comprehensively explore key metabolites and their correlation with diseases, and has a high degree of consistency with the results of targeted metabolomics validations [25]. Therefore, we believe that targeting the gut microbiota to enrich *A. muciniphila* to regulate host lipid metabolism may be an important target for SJP in the treatment of SCAD.

**Fig. 8:**
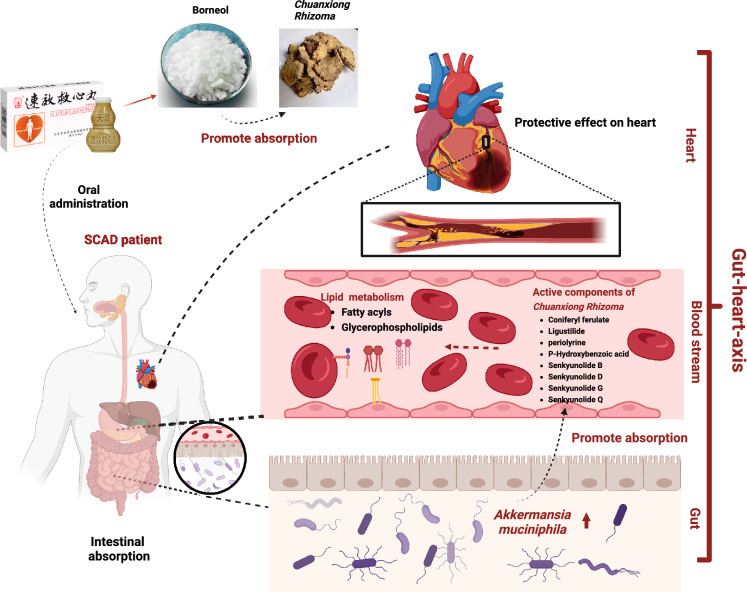
Diagram of the possible compatibility mechanism of SJP in the treatment of SCAD patients

In conclusion, this study revealed that the active components of SJP, *Chuanxiong Rhizoma* and borneol, may exert cardioprotective effects against SCAD through synergistic actions involving multiple pathways and multiple components. On the one hand, borneol enriches *A. muciniphila*, which regulates lipid metabolism, affects intestinal permeability, promotes the absorption and enrichment of active components of *Chuanxiong Rhizoma* into the blood, and enhances the efficacy of the effective components of *Chuanxiong Rhizoma*. On the other hand, borneol directly improves platelet function by regulating host lipid metabolism through the enrichment of *A. muciniphila* (Fig. 8).

## Conclusion

Our study provides novel insight into the compatibility mechanism of SJP in the treatment of SCAD. Our data suggest that SJP treatment for SCAD is closely associated with improvements in lipid metabolism. The active components of SJP, *Chuanxiong Rhizoma* and borneol, can regulate host lipid metabolism by enriching *A. muciniphila*, which promotes the absorption of *Chuanxiong Rhizoma*’s active components into the blood and exerts therapeutic effects. On the other hand, borneol may also directly exert therapeutic effects by regulating host lipid metabolism through the enrichment of *A. muciniphila*. Our research highlights the significant role of *A. muciniphila* in the multicomponent synergistic process of SJP, and the probiotic *A. muciniphila* may be an important tool for the prevention and treatment of SCAD.

## Supplementary Information

Supplementary Material 1. Materials and methods for microbiome and metabolomics experiments.

Supplementary Material 2.

## Data Availability

The data used and/or investigated during the present study are available from the corresponding author upon reasonable request.

## Abbreviations

SJP: Suxiao Jiuxin pill
SCAD: Stable coronary artery disease
CVD: Cardiovascular disease
TCM: Traditional Chinese medicine
ABX: Antibiotic
AKK: *Akkermansia muciniphila*
Bor: Borneol
CR: *Chuanxiong Rhizoma*
DMs: Differential metabolites
GC–MS: Gas chromatography–mass spectrometry
LC–MS: Liquid chromatography–mass spectrometry
UPLC-QTOF-MS: Ultra-high performance liquid chromatography with quadrupole time-of-flight mass spectrometry
PCA: Principal component analysis
PCoA: Principal coordinate analysis
PLS-DA: Partial least squares discriminant analysis
QC: Quality control
