# Supplementary material for: Integrated metabolomics and the microbiome reveal the compatibility mechanism of the Suxiao Jiuxin pill in the treatment of stable coronary artery disease
Source: Chin Med. 2025 Sep 1;20:140. doi: 10.1186/s13020-025-01198-8 (PMC12400554; doi:10.1186/s13020-025-01198-8)
Supplement: Supplementary file 1 — Supplementary Material 1. Materials and methods for microbiome and metabolomics experiments. [file 13020_2025_1198_MOESM1_ESM.docx]

**Integrated metabolomics and the microbiome reveal the compatibility mechanism of the Suxiao Jiuxin pill in the treatment of stable coronary artery disease**

Wanqi Le^1, †^, Jingyu Liao^1, †^, Yuhao Zhang^1^, Jingjing Xu^1^, Yuanyuan Zeng^2^, Houkai Li^3^, Xiaoxu Shen^2,^ *, Gaosong Wu^3,^ *, Weidong Zhang^1, 4, 5,^ *

^1^ Institute of Interdisciplinary Integrative Medicine Research, Shanghai University of Traditional Chinese Medicine, Shanghai, 201203, China.

^2^ Dongzhimen Hospital Affiliated to Beijing University of Chinese Medicine, Beijing, 100700, China

^3^ School of Pharmacy, Shanghai University of Traditional Chinese Medicine, Shanghai, 201203, China.

^4^ School of Pharmacy, Naval Medical University, Shanghai, 200433, China.

^5^ Institute of Medicinal Plant Development, Chinese Academy of Medical Sciences & Peking Union Medical College, Beijing 100193, China.

***Address for Corresponding authors**

**Weidong Zhang**

**Address:** No. 1200 Cai Lun Road, Pudong New District, Shanghai, China; No. 325 Guo He Road, Yangpu District, Shanghai, China. Fax and telephone: +86-21-51322770; E-mail: [wdzhangy@hotmail.com](mailto:wdzhangy@hotmail.com).

**Gaosong Wu**

**Address:** No. 1200 Cai Lun Road, Pudong New District, Shanghai, China. Fax and telephone: +86-21-51322171; E-mail: [gaosong@shutcm.edu.cn](mailto:gaosong@shutcm.edu.cn).

**Xiaoxu Shen**

**Address:** No. 5 Haiyuncang, Dongcheng District, Beijing, 100700, China.

**E-mail:** [jhxiaoxushen@163.com](mailto:jhxiaoxushen@163.com)

^†^These authors contributed equally.

**Supplementary Materials**

**1 Clinical indicators of SJP efficacy evaluation**

In order to evaluate the efficacy of SJP in the treatment of SCAD patients. The 14 identified symptoms of the patients were divided into three levels: main, secondary, and assistant indices (Supplementary Table 4). All symptoms were assigned different scores based on the severity of the symptoms (no symptoms = 0, mild symptoms = 1, moderate symptoms = 2, and severe symptoms = 3). The symptom score for each patient was summed to produce the final score for each patient before and after treatment.

**Table S1** Clinical symptom indexes for evaluating SJP treatment of SCAD patients.

| Indexes | Symptom | Symptom number |
| --- | --- | --- |
| Major index | Chest pain or precordial pain | S1 |
|  | Chest distress | S2 |
|  | Short breath | S3 |
|  | Palpitation | S4 |
|  | Chilly | S5 |
|  | Cold-limbs | S6 |
| Secondary index | Cold induced pain or exacerbation | S7 |
|  | Fullness in chest and hypochondrium | S8 |
|  | White complexion | S9 |
|  | White tongue coating | S10 |
|  | Pulse sinking late or tight | S11 |
| Assistant index | Night or cold tendon contracture pain | S12 |
|  | Joint cold pain | S13 |
|  | Lipnail pale or purple | S14 |

**2 Sample preparation**

50 μL of serum sample was deproteinized with 150 μL of MeOH in a 1.5 mL conical tube. After vortex mixed for 60 s and sonication for 10 min in an ice bath, samples were overnight at −20 °C to improve protein precipitation and then centrifuged at 12000 rpm for 15 min at 4 °C, 2 μL of supernatant was subjected to LC-MS analysis.

**3 Details of mass spectrometry parameter set**

Mass spectrometry detection was performed on the SYNAPT G2-Si HDMS system, equipped with an electrospray ionization source. Data acquisition progressed in both positive and negative ionization modes through MS and fast DDA acquisition mode. Mass spectrometry conditions were finally set as follows: dry gas flow rate, 900 L/h; dry gas temperature, 500 ℃; ion source temperature, 120 ℃; capillary voltage, 2.2 kV in positive ion mode and 2 kV in negative ion mode; cone voltage, 20 V, source offset, 80 V; cone gas flow, 50 L/h. The parameters in MS mode were set as follows: mass scan range, m/z 50-1200 Da; MS scan rate, 0.2 s. The parameters in fast DDA mode were set as follows: mass scan range, 50-1200 Da, MS scan rate, 0.2 s; the maximum number of ions for MS/MS from a single MS scan, 5; dual-dynamic MS/MS collision energies,10-40 V for low mass collision energy and 40-120 V for high mass collision energy. Real-time data was calibrated using an external reference (LockSprayTM) by the constant infusion of a leucine-enkephalin solution, with the lock masses at m/z 556.2771 in positive ion mode and m/z 554.2615 in negative ion mode, respectively, at a flow rate of 10 μL/min. Data acquisition was obtained by MassLynx V4.1.

**4 Data processing and analysis**

The raw MS data files were imported to the Progenesis QI (Waters, Milford, MA, USA) for data preprocessing including alignment, peak picking, and deconvolution to obtain the peak area list and the identification result list. Then signal drift of the deconvolution data preprocessed by Progenesis QI was corrected with statTarget (R package: R 3.6.3). Metabolites were selected as biomarker candidates on the following criteria: student's t-test, *p*-value < 0.05; OPLS-DA model, VIP-plots > 1. Then several online databases, such as the HMDB (<http://www.hmdb.ca/>), LIPID MAPS (<http://www.lipidmaps.org/>), and KEGG (<http://www.kegg.jp/>) were used to align the molecular mass data to identify metabolites using the corrected data files with exact mass measurement. The differential metabolites were uploaded to MetaboAnalyst 6.0 (<https://www.metaboanalyst.ca/>) and mapped into their biochemical pathways by the “Enrichment Analysis” module.

**Supplementary** **diagrams**


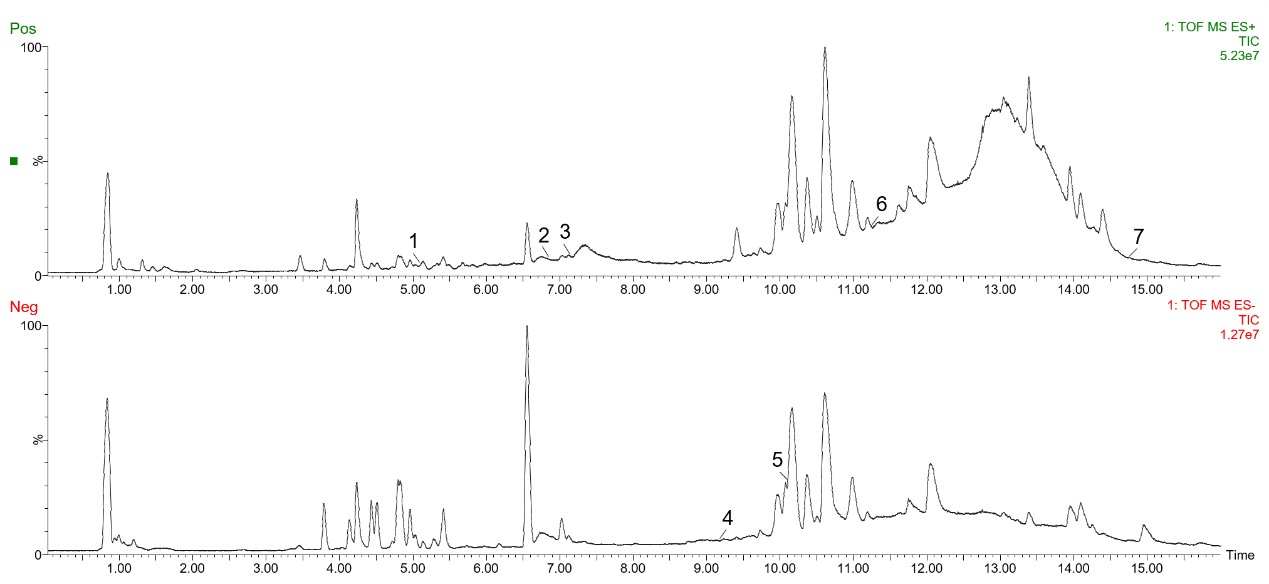


**Fig. S1.** TIC plot in positive and negative ion modes.


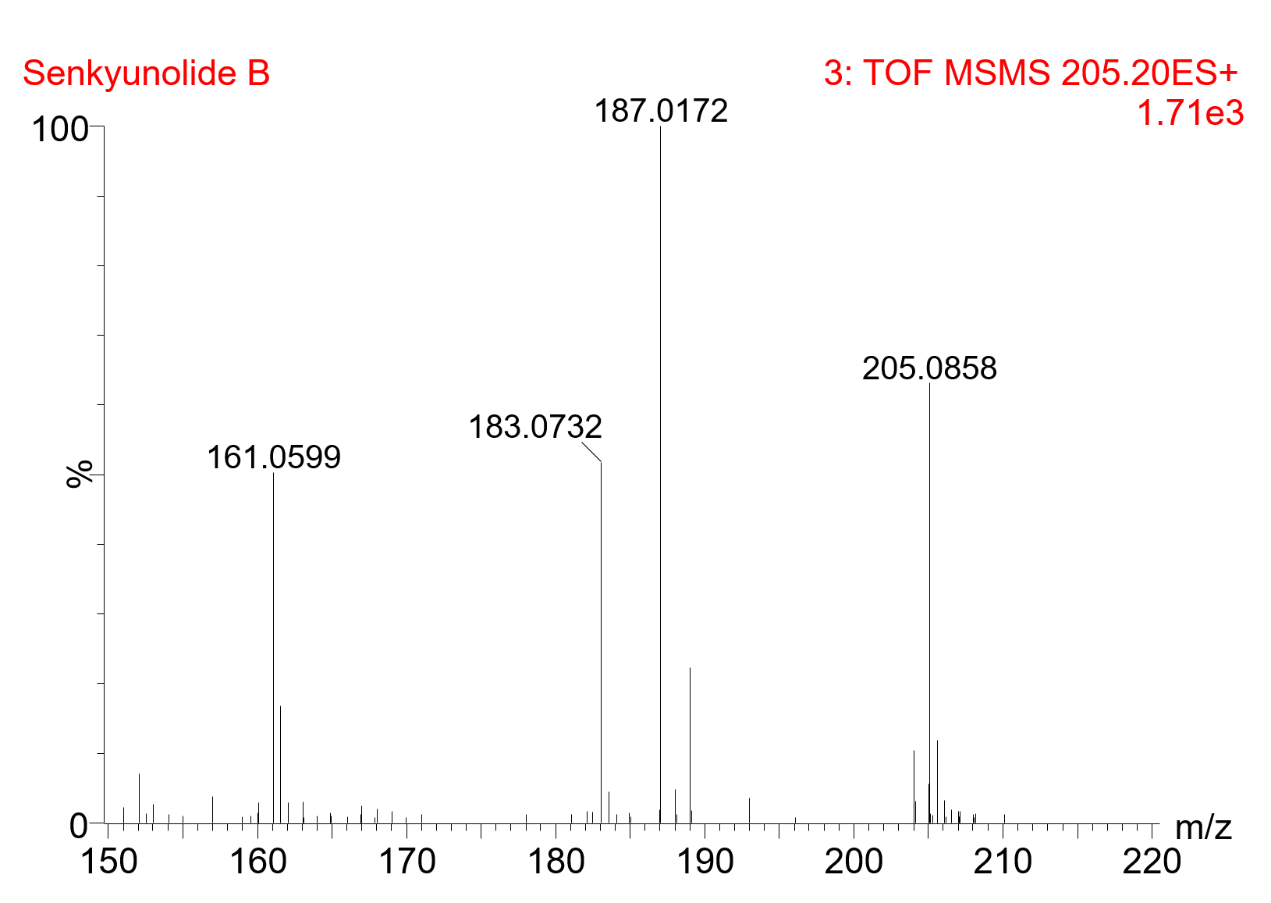


**Fig. S2.** MS/MS spectrum of Senkyunolide B.


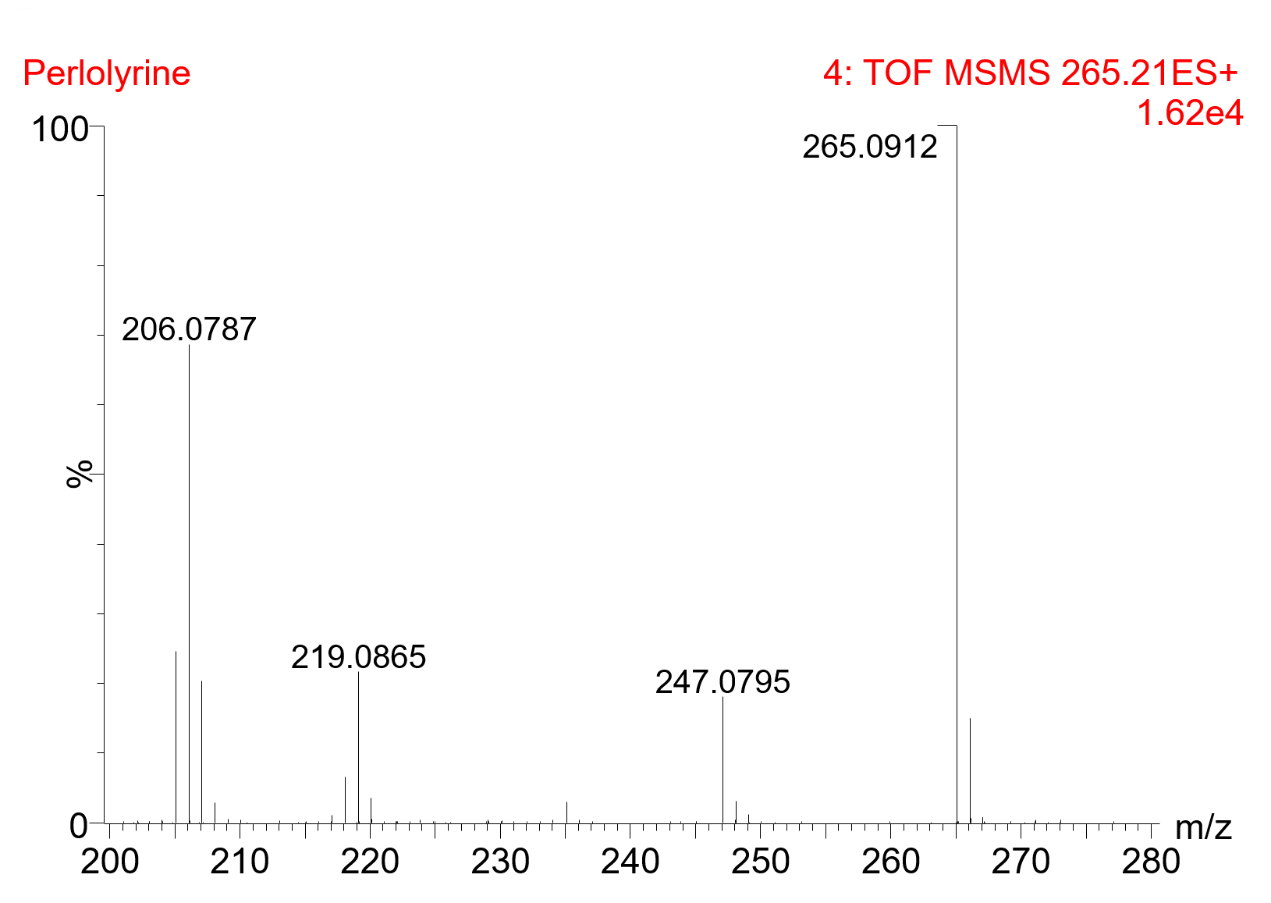


**Fig. S3.** MS/MS spectrum of Perlolyrine.


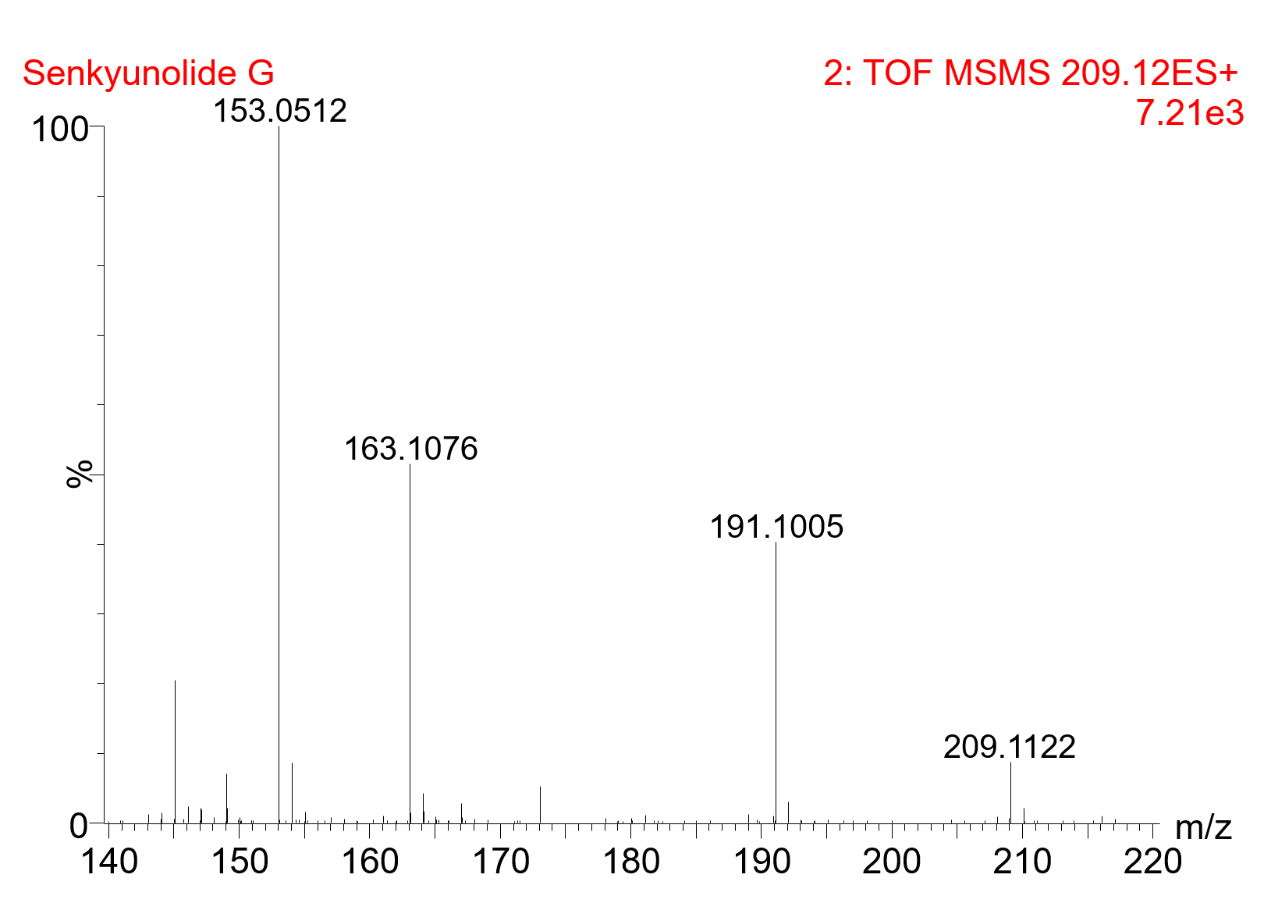


**Fig. S4.** MS/MS spectrum of Senkyunolide G.


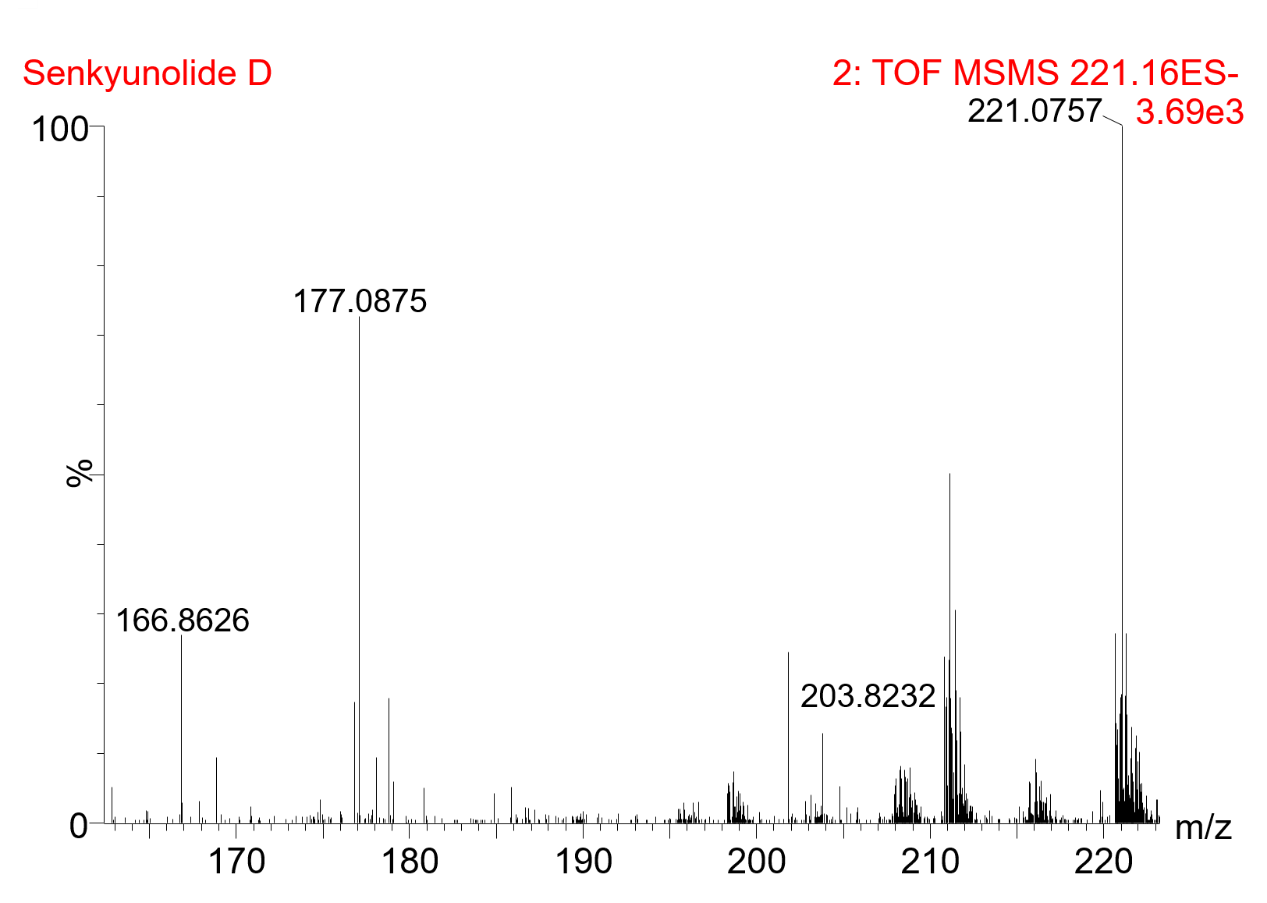


**Fig. S5.** MS/MS spectrum of Senkyunolide D.


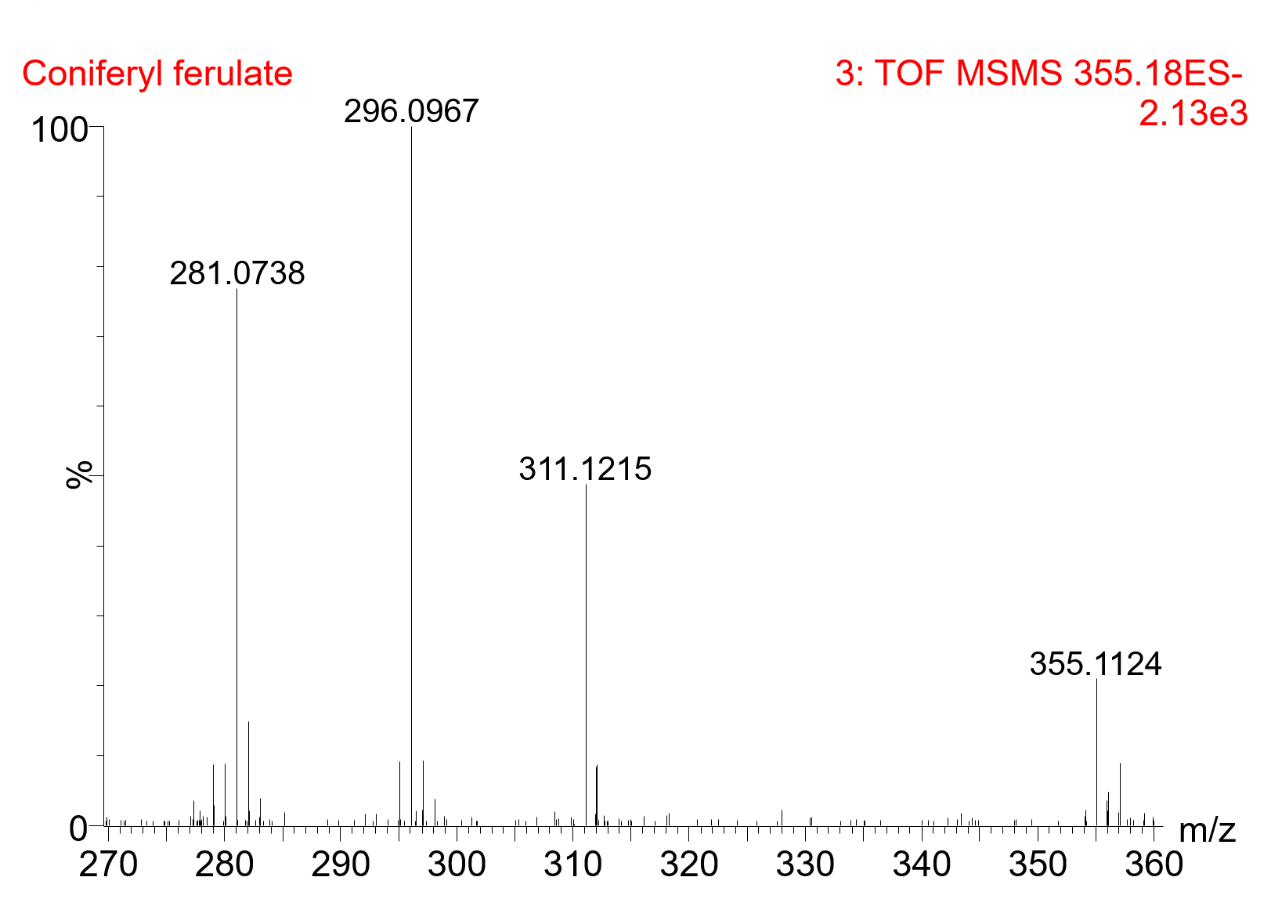


**Fig. S6.** MS/MS spectrum of Coniferyl ferulate.


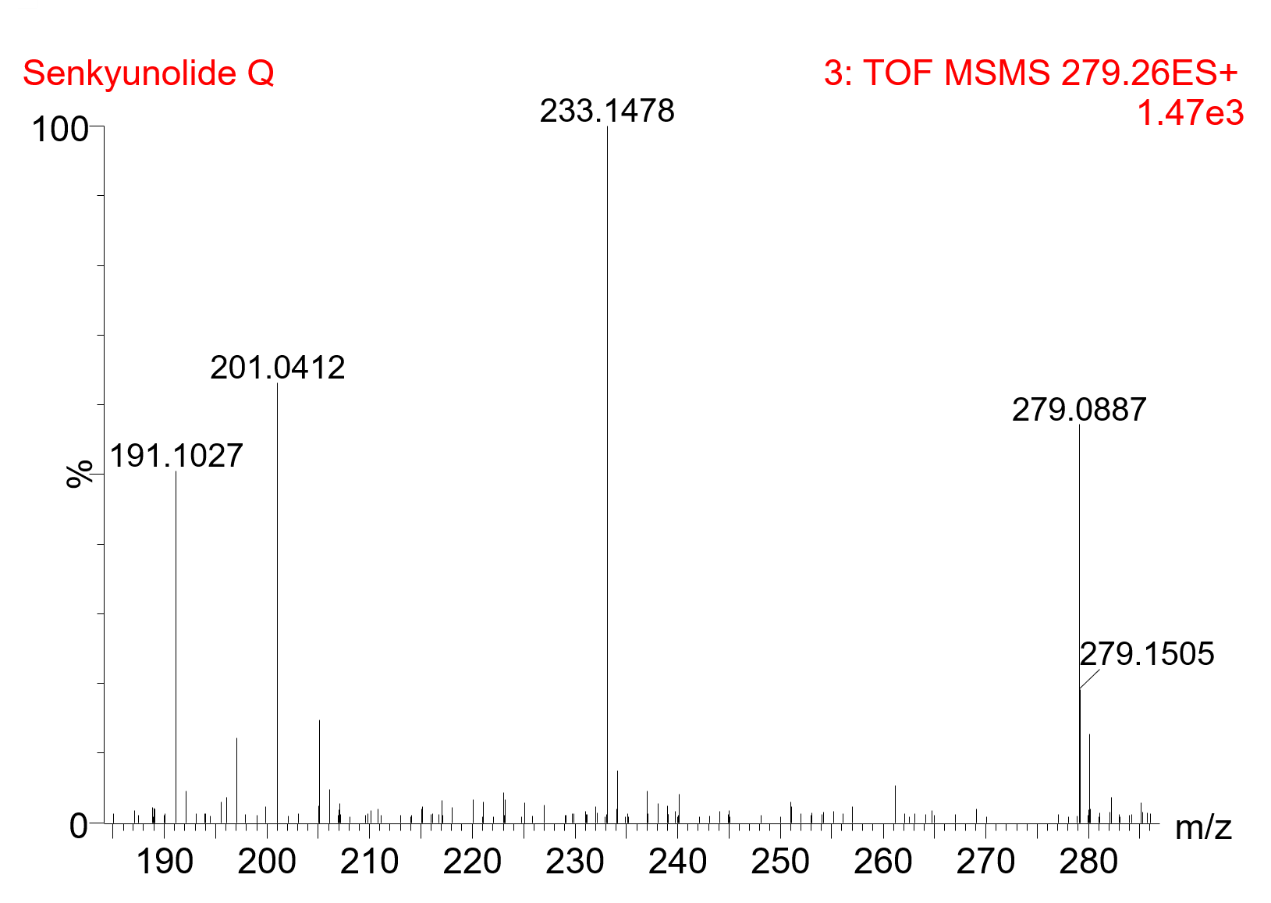


**Fig. S7.** MS/MS spectrum of Senkyunolide Q.


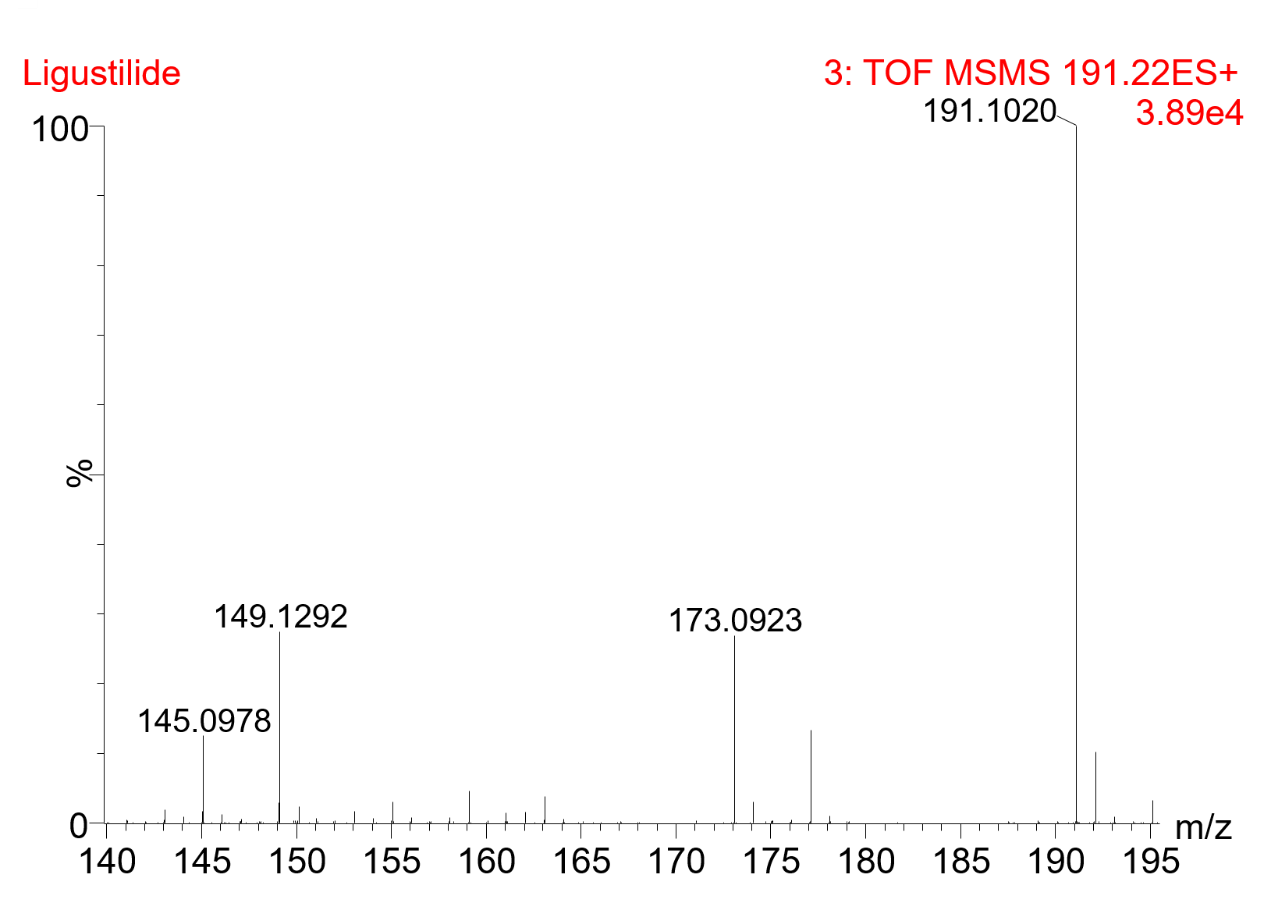


**Fig. S8.** MS/MS spectrum of Ligustilide.

**
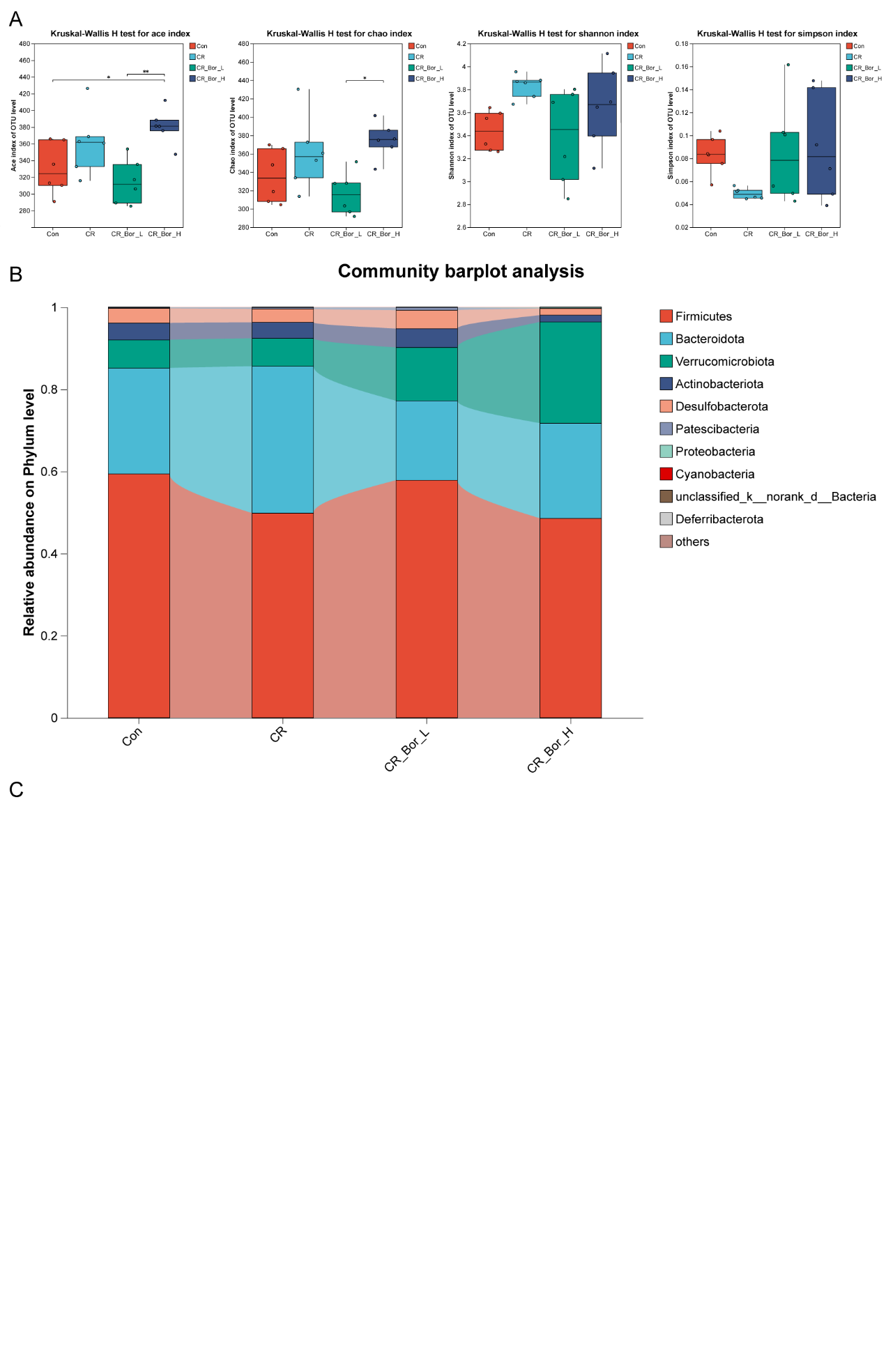
**

**Fig. S9.** (A) Species diversity differences between the Con, CR, CR_Bor_L, and CR_Bor_H groups were estimated by the observed ACE, Chao, Shannon, and Simpson indexes. (B) Gut microbiome structural analysis at the phylum level. Con: control group; CR: *Chuanxiong Rhizoma* treatment group; CR_Bor_L: *Chuanxiong Rhizoma* and low dose borneol treatment group; CR_Bor_H: *Chuanxiong Rhizoma* and high dose borneol treatment group, *n* = 6 per group.


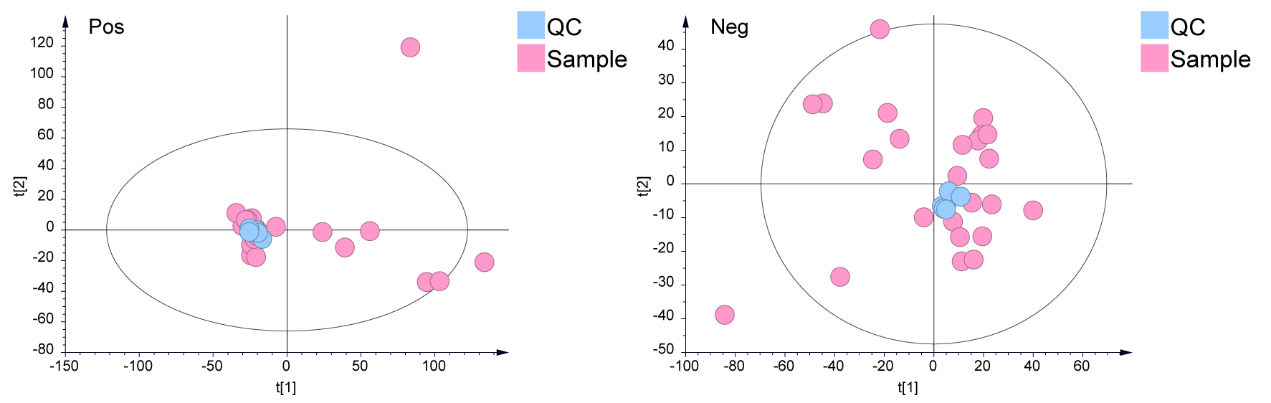


**Fig. S10.** PCA score plot of untargeted metabolome for serum sample in positive and negative mode.
